# Antibacterial Potential of Honeybee Venom and *Monascus purpureus* Extracellular Metabolites Against Multidrug-Resistant Pathogenic Bacteria

**DOI:** 10.3390/biology14010021

**Published:** 2024-12-28

**Authors:** Islam I. Teiba, Yasser S. A. Mazrou, Abeer H. Makhlouf, Yasser Nehela, Abdallah E. Mohamed, Ahmed M. Abbas, Islam Mamdouh, Emad H. El-Bilawy

**Affiliations:** 1 Department of Agricultural Botany, Faculty of Agriculture, Tanta University, Tanta 31527, Egypt; 2 Business Administration Department, Community College, King Khalid University, Guraiger, Abha 62529, Saudi Arabia; 3 Department of Agricultural Botany, Faculty of Agriculture, Minufiya University, Shibin El-Kom 32511, Egypt; 4 Land and Water Technologies Department, Arid Lands Cultivation Research Institute, City of Scientific Research and Technological Applications (SRTA-City), New Borg El-Arab 21934, Alexandria, Egypt; 5 Department of Microbiology and Immunology, Faculty of Pharmacy, Ain Shams University, African Union Organization St. Abbassia, Abbassia, Cairo 11566, Egypt; 6 Department of Microbiology & Immunology, Faculty of Pharmacy, King Salman International University (KSIU), Ras Sudr 46612, South Sinai, Egypt; 7 Faculty of Basic Sciences, King Salman International University (KSIU), Ras Sudr 46612, South Sinai, Egypt

**Keywords:** antimicrobial resistance, honeybee venom, metabolites, *Monascus purpureus*, *Escherichia coli*, *Staphylococcus aureus*, *Enterococcus faecalis*

## Abstract

Antimicrobial resistance (AMR) poses a critical global health threat, driving the search for alternative treatments to conventional antibiotics. In this study, the antibacterial properties of honeybee venom (BV) and fungal *Monascus purpureus* red dye (RD) were evaluated against three multidrug-resistant bacterial pathogens. Extracts of BV and RD exhibited dose-dependent antibacterial activity against the three tested bacteria, with their strongest effectiveness against *S. aureus* (minimum inhibitory concentrations [MIC] = 3.18 and 6.315 μg·mL^−1^, respectively). Although the three bacterial strains were resistant to the antibiotic ampicillin-sulbactam (10/10 µg), both extracts exhibited superior antibacterial activity against the three bacterial strains compared to five standard antibiotics, especially RD extract, which produced the largest inhibition zone (20 ± 0.20 mm) against *S. aureus.* The larger inhibition zones against *S. aureus* suggest its high sensitivity, whereas *E. coli* and *E. faecalis* exhibited smaller inhibition zones, indicating less sensitivity to BV and RD extracts. Differences in the inhibition zones suggest the variations in antimicrobial activity between the two extracts and their strain-specific effectiveness. Scanning electron microscopy (SEM) revealed that BV and RD extracts disrupted the bacterial plasma membrane, suggesting that the bioactive compounds penetrate the bacterial cell wall and alter its integrity. Furthermore, GC–MS-based analysis revealed that the chemical composition of BV and RD extracts exhibited highly diverse structures, including complex polycyclic systems, porphyrins, steroids, and esters. For instance, 42 metabolites were identified in the BV extract, which mainly were organic and metal–organic compounds; however, only 23 molecules were identified in RD extract, which mainly were fatty acids and their derivatives. The diversity in the chemical compositions of both extracts highlights their potential applications in pharmaceuticals, materials, and biochemistry fields. Collectively, these findings indicate that honeybee venom and the red dye from *M. purpureus* have promising antibacterial properties and warrant further investigation as potential alternatives to conventional antibiotics. Further multi-ligand docking-based virtual screening studies are required to identify the most promising detected metabolite(s) within both BV and RD extracts.

## Simple Summary

This study explores and develops eco-friendly alternatives for multidrug-resistant bacterial pathogens. Herein, the potential antibacterial activity of honeybee venom (BV) and *Monascus purpureus* red dye (RD) extracts was investigated. Both BV and RD extracts inhibited the bacterial growth of *E. coli*, *S. aureus*, and *E. faecalis* in a dose-dependent manner while being more effective against *S. aureus* (MIC = 3.18 and 6.315 µg·mL^−1^, respectively). It is worth mentioning that the highest concentration (200 μg·mL^−1^) of both extracts was more effective than several standard antibiotics, particularly RD, which produced a significant inhibition zone (~20 mm) against *S. aureus*. SEM-based microscopy showed that both extracts disrupt bacterial cell membranes, indicating their ability to penetrate and damage bacterial cell walls. Moreover, GC-MS-based analysis showed high diversity in BV’s chemical composition and bioactive compounds (42 metabolites) and RD (23 metabolites), including polycyclic systems, fatty acids, and esters. Collectively, these findings suggest that BV and RD have strong antibacterial properties and potential applications as alternatives to conventional antibiotics. However, supplementary investigations are required to better understand these extracts’ physio-biochemical and molecular mechanisms within treated bacterial cells.

## 1. Introduction

Antimicrobial resistance (AMR) is a significant challenge predicted by the World Health Organization (WHO) as a “global public health concern” [1]. It has serious global implications, leading to higher rates of illness and death from bacterial infections, and urgent measures are needed to combat this issue [2]. Over the last 30 years, the rate of new antibiotic approvals has declined, while antibiotic-resistant bacterial pathogens continue to emerge [3]. These pathogens are increasingly developing additional resistance mechanisms, leading to the rise of multidrug-resistant (MDR), extensively drug-resistant (XDR), and pan-drug-resistant (PDR) bacteria that resist all known antibiotics [4]. AMR poses a worldwide challenge, impacting the health of both humans and animals. While the primary cause of AMR is the overuse of antibiotics and antimicrobials, it is also fueled by poor sanitation, pollution, and other non-medical factors, with the natural environment significantly contributing to its spread [5,6]. The AMR phenomenon accounted for 1.27 million deaths in 2019 globally, and it is projected to cause over 10 million deaths by 2050. In 2020, the WHO warned that without changes in antibiotic usage, AMR could gradually lead to a pandemic [6]. In 2019, Egypt recorded 16,100 deaths directly attributable to AMR, along with an additional 56,600 deaths associated with it, ranking 58th in terms of age-standardized mortality rate per 100,000 populations among 204 countries [7].

In the context of antimicrobial resistance, the complex web of microbial interactions within ecosystems can be disrupted, exacerbating the spread of resistant pathogens [8]. Microorganisms, found throughout the biosphere, play vital roles in shaping their surroundings, with effects that can be beneficial, harmful, or subtle [9]. While many bacteria contribute positively to human health and other organisms, certain species are responsible for a wide array of diseases in humans and animals, causing illnesses that range from mild to life threatening. For instance, methicillin-resistant *Staphylococcus aureus* (MRSA) is a notable example of an AMR strain. *Staphylococcus aureus* is a noteworthy bacterial human pathogen in humans, accountable for a wide range of clinical conditions [10]. This Gram-positive coccal bacterium typically forms clusters [11]. *S. aureus* is one of the most prevalent bacterial pathogens in humans, causing various infections, including bacteremia, infective endocarditis, skin and soft tissue infections, gastroenteritis, meningitis, toxic shock syndrome, and urinary tract infections [12]. Infections with *S. aureus* occur frequently in both community and hospital settings, and treatment is complicated by the rise of multi-drug-resistant strains [13].

Likewise, AMR in *Escherichia coli* is a growing concern. *E. coli* has a complex relationship with its host [14]. While some strains harmlessly coexist in the intestines, aiding in digestion [15], others can cause serious systemic infections [16]. Genetic diversity among *E. coli* strains means that some possess virulence factors that enable them to cause diseases in various species. Pathogenic *E. coli* can produce toxins that disrupt the digestive system, resulting in symptoms like cramps, diarrhea, and vomiting [17]. Food contamination is a common route for these harmful strains, emphasizing the need for good food-handling practices to prevent *E. coli* infections [18].

Another example of AMR human pathogenic bacteria is the vancomycin-resistant *Enterococcus faecalis* (VRE). *E. faecalis* is a human pathobiont that can exhibit both commensal and pathogenic behaviors [19]. Its pathogenicity is often linked to host vulnerability, excessive intestinal growth, or the presence of medical devices [20]. Traditionally viewed as an extracellular pathogen, *E. faecalis* can adhere to and invade various mammalian cells, though not as effectively as typical intracellular pathogens [21]. The adhesion of *E. faecalis* to epithelial cells is likely mediated more by carbohydrate structures than by protein components [22]. *E. faecalis* is responsible for a significant portion of enterococcal infections and is mainly associated with urinary tract infections (UTIs), bloodstream infections, and endocarditis (inflammation of the heart valves) [19]. Some strains of *E. faecalis* are concerning due to their antibiotic resistance, complicating treatment options [23]. The cases of these three bacterial species underscore the alarming rise in antibiotic resistance and highlight the urgent need for improved hygiene and food-handling practices to prevent infections. Growing concerns over antibiotic overuse have led to increased interest in eco-friendly alternatives derived from biological sources [24]. Honeybee venom (BV) and *Monascus* fermentation products might be promising alternatives.

Honeybee venom (BV) is a promising therapeutic candidate, known for its health benefits across various organisms. Traditionally used as a natural pain reliever and anti-inflammatory agent, honeybee venom contains over 40 bioactive substances, including peptides, enzymes, and other compounds that contribute to its medicinal properties [25]. Key components such as melittin, apamin, and adolapin are believed to play significant roles in their anti-inflammatory, antibacterial, and pain-relieving effects [26]. Additionally, some compounds, like apamin and phospholipase A2, show potential for treating immune disorders through their immune-regulating effects [27]. BV’s therapeutic advantages extend to veterinary medicine, benefiting both livestock and companion animals [28]. Similarly, *Monascus* pigments, derived from species such as *Monascus purpureus*, are popular as natural food colorants in East Asian countries, where they enhance both color and sensory quality [29]. Due to increasing interest in natural products, *Monascus purpureus* has been widely studied for its bioactive compounds, including flavonoids, phenols, tannins, and various secondary metabolites. Research suggests that fermenting rice with *Monascus purpureus* may offer additional health benefits, such as cholesterol regulation, diabetes management, cardiovascular support, and possibly even cancer prevention [30,31].

Antimicrobial resistance (AMR) is a serious global health challenge, yet sustainable alternatives to conventional antibiotics remain poorly studied. Although both honeybee venom (BV) and *Monascus purpureus* red dye (RD) have been recognized for their medicinal and antimicrobial properties, their comparative effectiveness against multidrug-resistant (MDR) human pathogenic bacteria is poorly studied. Moreover, the exact cellular mechanisms behind their activity, particularly their effects on bacterial membranes and the role of specific metabolites, remain largely unexplored. This study hypothesizes that BV and RD extracts exhibit differential antibacterial activity against common MDR human bacterial pathogens such as *E. coli*, *S. aureus*, and *E. faecalis*. Moreover, we aimed to evaluate their antibacterial activity compared to traditional antibiotics, investigate their mode of action using SEM imaging, characterize their chemical composition through GC–MS, and correlate dominant metabolites with potential antimicrobial activity. By investigating the antimicrobial mechanisms of these two natural products, this research aims to provide insights that could inform the development of alternative antimicrobial strategies to combat the growing challenge of antibiotic resistance.

## 2. Materials and Methods

### 2.1. Source and Extraction of Honeybee Venom

The honeybee venom (BV) used in this study was sourced from honeybee (*Apis mellifera*) colonies maintained at the apiary of Tanta University’s Faculty of Agriculture. Venom was collected using a semi-automatic extraction device consisting of stainless-steel electrodes connected to a battery and pulse generator, along with a glass collection slide. To extract the venom, the bees were stimulated with controlled electrical impulses, which prompted them to sting the glass collection surface. The venom secreted by the bees was deposited on the glass slide, and the volatile components were allowed to evaporate, leaving behind a white residue of crude honeybee venom. This sediment was carefully scraped off the glass slide and stored at 4 °C before further use. For the experiments, 1 mg of the collected honeybee venom was dissolved in 1 mL of dimethyl sulfoxide (DMSO) to prepare a 1000 μg·mL^−1^ stock solution. This stock solution was used to prepare the desired concentrations of honeybee venom for the antimicrobial assays.

### 2.2. Source and Extraction of Red Dye

The *Monascus purpureus* strain ATCC 16436 used in this study was obtained from the Microbiological Resources Center (MIRCEN) in Cairo, Egypt, which is part of the Egyptian National Culture Collection (ENCC). This specific strain is known for its ability to produce red pigments. For activating and short-term preservation of the fungal strain, potato dextrose agar (PDA) was used. To prepare the modified minimal medium, as described in previous studies [32,33], 4 mL of the medium was mixed with 3.0 g of rice grains per 100 mL. The composition of the modified medium included 0.5 g each of NH_4_SO_4_, NH_4_NO_3_, KNO_3_, and peptone, along with 0.2 g of KH_2_PO_4_ and K_2_HPO_4_, 0.2 g of MgSO_4_, 0.001 mM ZnSO_4_, and 0.002 mM MnSO_4_. This modified medium was the primary medium used for the biosynthesis of *Monascus* pigments, with the final pH adjusted to 4.5. A fungal plug was transferred to the PDA plates and then incubated at 30 °C for 7 days. Subsequently, the inoculated plates were used to prepare a 2 × 10^4^ spores·mL^−1^ fungal spore suspension using sterile water.

For the extraction of *Monascus* pigments, a slightly modified method from previous studies was employed [34]. After the 7-day incubation period, 20 mL of 96% ethanol was added to the fermented rice substrate, and the mixture was agitated at 180 rpm for 2 h. The mixture was then filtered through Whatman filter paper No. 1. The filtrate was centrifuged at 10,000 rpm for 10 min, and the resulting supernatant containing the *Monascus* red pigments was collected and stored at 4 °C for further use. The alcohol trace was eliminated by volatilization under vacuum at room temperature. To prepare the extract for antibacterial activity testing, 1 mg of the concentrated *Monascus* red pigment was dissolved in 1 mL of DMSO to obtain a 1000 μg·mL^−1^ stock solution.

### 2.3. Chemical Profiling of the Honeybee Venom and Red Dye Extracts

The chemical compositions of the honeybee venom and *Monascus* red dye extracts were analyzed using gas chromatography–tandem mass spectrometry (GC–MS/MS). The GC–MS/MS analysis was conducted with the following parameters: column oven temperature initially set to 50 °C, then increased by 5 °C/min to 250 °C and held for 2 min, further increased to a final temperature of 300 °C at a rate of 30 °C/min and maintained for another 2 min; injector and MS transfer line temperatures of 270 °C and 260 °C, respectively; helium carrier gas at a constant flow rate of 1 mL per minute; 4-min solvent delay; and 1 μL aliquot of the diluted samples injected automatically in split mode using an autosampler AS1300 (Thermo Fisher Scientific Inc., Rodano-Milan, Italy). An MS detector (Perkin Elmer, Waltham, MA, USA) was set to electron ionization (EI) at 70 eV, scanning 50–650 *m*/*z* in full scan mode with 200 °C as the ion source temperature. GC–MS chromatograms of honeybee venom and *Monascus* red dye extracts were analyzed, and detected peaks were identified by comparing their mass spectra with library entries of NIST-14 and Wiley, 9th edition.

### 2.4. Antibacterial Activity

#### 2.4.1. Bacterial Strains

The antibacterial properties of BV and RD extracts were evaluated using three reference bacterial strains, *Escherichia coli* ATCC 8739, *Staphylococcus aureus* ATCC 6538, and *Enterococcus faecalis* ATCC 25923, obtained from the Global Bioresource Center (ATCC, Manassas, VA, USA). These strains were selected, as they represent standard reference organisms widely used in antimicrobial susceptibility testing and serve as essential baseline controls for comparing novel antimicrobial agents [35]. *E. coli* was chosen as a representative Gram-negative organism, while *S. aureus* and *E. faecalis* represent Gram-positive bacteria. These well-characterized strains provide a foundational framework for understanding the antimicrobial mechanisms of BV and RD, establishing a methodological baseline for future studies with resistant strains such as MRSA.

#### 2.4.2. Determination of Minimum Inhibitory Concentration (MIC) and the Half-Maximal Inhibitory Concentration (IC_50_)

The minimum inhibitory concentration (MIC) and the half-maximal inhibitory concentration (IC_50_) were determined using the Clinical and Laboratory Standards Institute (CLSI) method [36] with 96-well microplates. Briefly, a bacterial inoculum of 1.5 × 10^8^ CFU·mL^−1^ was introduced into each well. Different concentrations of the BV and RD extracts were prepared in DMSO (200, 100, 50, 25, 12.5, 6.25, 3.125, 1.562, 0.781, and 0.390 μg·mL^−1^) and tested against the three bacterial strains in Mueller–Hinton broth (Oxoid, Basingstoke, Hampshire, UK). For each test, three wells containing bacterial suspension with solvent (DMSO), but without the extract served as growth controls, while three additional wells without bacterial inoculum acted as background controls. The optical density (O.D.) was measured at 620 nm using a microtiter plate reader (Thermo Scientific, Norristown, PA, USA). MIC (concentration that inhibited over 95% of bacterial growth) was calculated using a modified Gompertz function [37] using Equation (1) as follows:

MIC = 10^(M+[1/B])^ (1)

where B is a slope parameter and M is the log concentration of the inflexion point.

On the other hand, the half-maximal inhibitory concentration (IC_50_) was calculated via probit regression analysis.

#### 2.4.3. Comparison of the Antibacterial Activity of the Extracts and Traditional Antibiotics

The comparison was carried out using the disc diffusion method according to the protocol established by Surendra et al. [38]. Initially, the three bacterial strains (*E. coli*, *S. aureus*, and *E. faecalis*) were cultured in 1.5 mL of brain heart infusion broth (Oxoid, Basingstoke, Hampshire, UK), and then incubated at 37 °C for 24 h to prepare the pure bacterial cultures. Subsequently, the newly prepared bacterial cultures were streaked onto brain heart infusion agar medium and then incubated at 37 °C for an additional 24 h. A 0.5 McFarland standard (1.5 × 10^5^ CFU·mL^−1^) of each bacterial strain was prepared by transferring a proper amount of the three tested bacterial strains into 10 mL of sterile saline. Then, bacterial cultures were evenly streaked on Mueller–Hinton agar (MH, Oxoid, Basingstoke, Hampshire, UK) plates using a sterile cotton swab.

Cellulose discs (6.3 mm diameter) were infused with the BV and RD extracts at the highest concentration used in the study (200 μg·mL^−1^) and placed on the agar surface. The tests were performed in triplicate. After incubating for 24 h at 37 °C, the plates were examined, and the inhibition zones were measured to calculate the average inhibition zone. Additionally, antibiotic discs, including ciprofloxacin (CIP; 5 µg), azithromycin (AZM; 15 µg), streptomycin (S; 10 µg), ampicillin-sulbactam (A/S; 10/10 µg), and clarithromycin (CLR; 15 µg), were added to the plates. After 24-h incubation at 37 °C, the inhibition zones were measured, and the results were reported as the mean inhibition zone in millimeters. The interpretation of antibiotic susceptibility was based on the Clinical and Laboratory Standards Institute (CLSI) guidelines, allowing direct comparison between the extracts’ inhibition zones and standard antibiotic performance under identical experimental conditions.

#### 2.4.4. Cellular Structure of the Tested Bacterial Strains

To examine malformations in bacterial cellular structures, the tested bacterial strains were cultured in nutrient broth (NB) media (Merck Millipore, Darmstadt, Germany) with BV and RD extracts (200 μg·mL^−1^) and incubated at 37 °C for 12 h, alongside a non-treated control. After incubation, formalin–glutaraldehyde fixative (4F1G) in 0.1 M phosphate buffer (pH 7.4) was used to fix the bacteria after centrifugation. In the same buffer, the specimens were subsequently fixed once again using 1% osmium tetroxide. Following fixation, the specimens were dehydrated through a series of acetone concentrations. Then, they were coated with gold–palladium using a Polaron E500 sputter coater (Polaron Equipment Ltd., Hertfordshire, England). The bacterial specimens were analyzed using a scanning electron microscope (Model JSM 35C, JEOL Ltd., Akishima, Tokyo, Japan).

### 2.5. Statistical Analysis

The completely randomized design, unless otherwise stated, was used as the standard experimental design throughout this study. All experiments were conducted in triplicate, with three biological replicates for each treatment, and all tests were performed in triplicate. One-way ANOVA (Analysis of Variance) was used to test the statistically significant differences between treatments, followed by Tukey’s HSD for post hoc pairwise comparisons (*p* ≤ 0.05). The MIC was calculated using a modified Gompertz function [37], whereas the IC_50_ was calculated using probit regression analysis with 95% confidence intervals. The overall model fit of the probit regression model was stated using the chi-square (χ^2^), *p*-value, and coefficient of determination (R^2^) of Cox & Snell and Nagelkerke. To minimize variability across replicates, all experiments were conducted under controlled temperature conditions (±0.1 °C), using single batches of reagents, and following a standardized protocol with precise timing of sample preparation steps; each analysis was performed by the same researcher to eliminate inter-operator variation.

## 3. Results

### 3.1. Honeybee Venom (BV) Extract Exhibited Notable Antibacterial Activity Against MDR Human Pathogenic Bacteria

**Figure 1:**
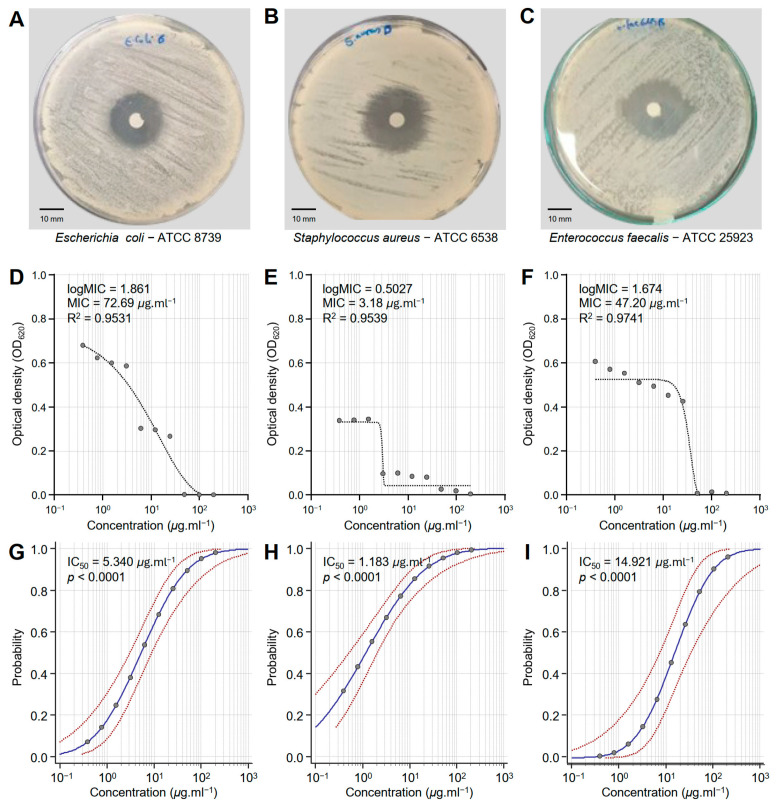
In vitro antibacterial activity of honeybee venom (BV) extracts against three multidrug-resistant human pathogenic bacteria. (**A**–**C**) Disc diffusion method of the BV extract (200 μg·mL^−1^) against *Escherichia coli*—ATCC 8739, *Staphylococcus aureus*—ATCC 6538, and *Enterococcus faecalis*—ATCC 25923, respectively. (**D**–**F**) Susceptibility analysis and minimum inhibitory concentrations (MICs) of BV extract against *E. coli*, *S. aureus*, and *E. faecalis*, respectively. Different concentrations of the BV extract were prepared in DMSO (200, 100, 50, 25, 12.5, 6.25, 3.125, 1.562, 0.781, and 0.390 μg·mL^−1^). (**G**–**I**) Probit regression (dose–response analysis) of BV extract against *E. coli*, *S. aureus*, and *E. faecalis*, respectively. Gray dots present the means of three replicates of each concentration. Blue solid lines represent the probit regression lines, whereas red dashed lines edge represent the 95% confidence intervals for the estimated regression. Probit-associated half-maximal inhibitory concentrations (IC_50_; μg·L^−1^), 95% confidence intervals, and overall model fit are listed in Table 1. For BV extract, the IC_50_ against *E. coli* ATCC8739 was 5.340 μg·mL^−1^ (95% CI: 3.080–8.929 μg·mL^−1^), against *S. aureus* ATCC 6538 it was 1.183 μg·mL^−1^ (95% CI: 0.524–2.069 μg·mL^−1^), and against *E. faecalis* ATCC 25923 it was 14.921 μg·mL^−1^ (95% CI: 7.181–32.692 μg·mL^−1^).

Honeybee venom (BV) extract exhibited significant antibacterial activity against *E. coli*—ATCC 8739 (Figure 1A), *S. aureus*—ATCC 6538 (Figure 1B), and *E. faecalis*—ATCC 25923 (Figure 1C). Although BV extract showed putative antibacterial activity against *E. coli* (logMIC = 1.861, MIC = 72.69 μg·mL^−1^, and R^2^ = 0.9531) (Figure 1D), it demonstrated its strongest effectiveness against *S. aureus* (logMIC = 0.5027, MIC = 3.18 μg·mL^−1^, and R^2^ = 0.9539) (Figure 1E). Moreover, BV extract displayed comparable antibacterial activity against *E. faecalis* (logMIC = 1.674, MIC = 47.20 μg·mL^−1^, and R^2^ = 0.9741) (Figure 1F). It is worth mentioning that the probit regression (dose–response analysis) of honeybee venom (BV) extract against the three bacterial strains (*E. coli*, *S. aureus*, and *E. faecalis*) (Figure 1G, Figure 1H and Figure 1I, respectively) exhibited strong overall high chi-square (χ^2^) values and significant *p*-values (*p* < 0.0001) (Table 1). *S. aureus* is the most sensitive bacterial strain (IC_50_ = 1.183 μg·mL^−1^), followed by *E. coli* (IC_50_ = 5.340 μg·mL^−1^), and *E. faecalis* as the least sensitive (IC_50_ = 14.921 μg·mL^−1^). These findings highlight the potential of BV extract as a promising antibacterial alternative across different pathogens, as well as its potential for targeted applications.

### 3.2. Monascus Red Dye (RD) Extract Exhibited Strong Antibacterial Activity Against MDR Human Pathogenic Bacteria

**Figure 2:**
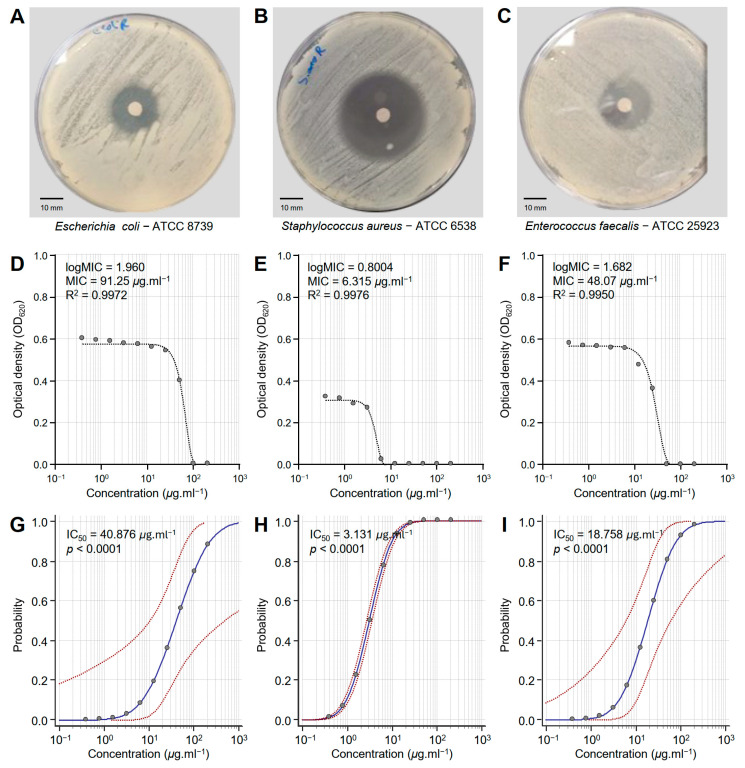
In vitro antibacterial activity of *Monascus* red dye (RD) extracts against three multidrug-resistant human pathogenic bacteria. (**A**–**C**) Disc diffusion method of the RD extract (200 μg·mL^−1^) against *Escherichia coli*—ATCC 8739, *Staphylococcus aureus*—ATCC 6538, and *Enterococcus faecalis*—ATCC 25923, respectively. (**D**–**F**) Susceptibility analysis and minimum inhibitory concentrations (MICs) of RD extract against *E. coli*, *S. aureus*, and *E. faecalis*, respectively. Different concentrations of the RD extract were prepared in DMSO (200, 100, 50, 25, 12.5, 6.25, 3.125, 1.562, 0.781, and 0.390 μg·mL^−1^). (**G**–**I**) Probit regression (dose–response analysis) of RD extract against *E. coli*, *S. aureus*, and *E. faecalis*, respectively. Gray dots present the means of three replicates of each concentration. Blue solid lines represent the probit regression lines, whereas red dashed lines edge represent the 95% confidence intervals for the estimated regression. Probit-associated half-maximal inhibitory concentrations (IC_50_; μg·L^−1^), 95% confidence intervals, and overall model fit are listed in Table 1. For RD extract, the IC_50_ against *E. coli* ATCC8739 was 40.876 μg·mL^−1^ (95% CI: 10.632–491.037 μg·mL^−1^), against *S. aureus* ATCC 6538 it was 3.131 μg·mL^−1^ (95% CI: 2.729–3.592 μg·mL^−1^), and against *E. faecalis* ATCC 25923 it was 18.758 μg·mL^−1^ (95% CI: 6.201–61.248 μg·mL^−1^).

The susceptibility of three multidrug-resistant human pathogenic bacteria to different concentrations of the *Monascus* red dye (RD) extract was tested in vitro (Figure 2). RD extract showed strong antibacterial activity against *E. coli*—ATCC 8739 (Figure 2A), *S. aureus*—ATCC 6538 (Figure 2B), and *E. faecalis*—ATCC 25923 (Figure 2C). However, *S. aureus* was more susceptible to RD extracts than the other bacterial strains.

Susceptibility analysis showed that the minimum inhibitory concentration (MIC) of RD extract that was required to inhibit *E. coli* (logMIC = 1.960, MIC = 91.25 μg·mL^−1^, and R^2^ = 0.9972) was relatively high compared to other bacterial strains (Figure 2D). However, the dose–response curve of *S. aureus* demonstrated the highest sensitivity with an MIC of 6.315 μg·mL^−1^ (logMIC = 0.8004 and R^2^ = 0.9976) (Figure 2E). On the other hand, *E. faecalis* exhibited intermediate sensitivity to RD extracts with an MIC of 48.07 μg·mL^−1^ (logMIC = 1.682 and R^2^ = 0.9950) (Figure 2F). It is worth mentioning that all models were fitted with high R^2^ values (more than 0.99), highlighting the strong fit of the model and confirming the reliability of the predicted MIC.

Moreover, probit analysis showed the probability of RD extract to inhibit *E. coli* (Figure 2G), *S. aureus* (Figure 2H), and *E. faecalis* (Figure 2I). Briefly, *E. coli* showed a steep response curve, indicating that higher concentrations are required for effective inhibition (IC_50_ = 40.876, *p* < 0.0001), suggesting reduced sensitivity (Table 1 and Figure 2G). On the other hand, *S. aureus* exhibited a sharper transition and a more centered curve at lower concentrations (IC_50_ = 3.131, *p* < 0.0001), reflecting higher sensitivity (Table 1 and Figure 2H). Similarly, *E. faecalis* exhibited an intermediate sensitivity to RD extract, with the response curve shifted slightly towards higher concentrations (IC_50_ = 18.758, *p* < 0.0001) compared to *S. aureus* but relatively lower than *E. coli* (Table 1 and Figure 2I).

### 3.3. Antibacterial Activity of the BV and RD Extracts Against Traditional Antibiotics

**Figure 3:**
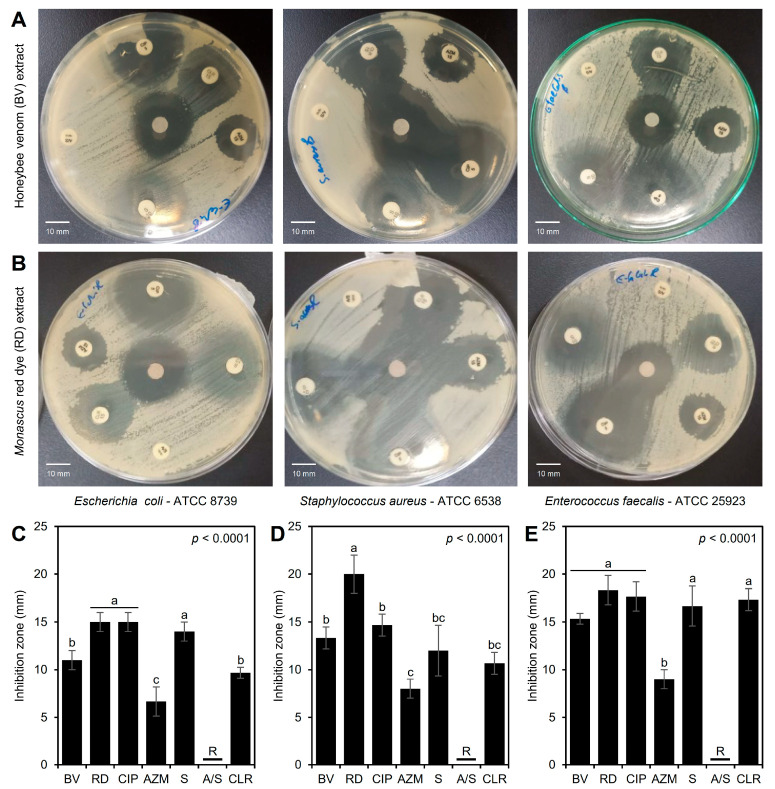
In vitro antibacterial activity of honeybee venom (BV) and *Monascus* red dye (RD) extracts against multidrug-resistant human pathogenic bacteria in comparison with traditional antibiotics. (**A**,**B**) Disc diffusion method of the BV and RD extract (200 μg·mL^−1^) compared with the traditional antibiotics against *Escherichia coli*—ATCC 8739, *Staphylococcus aureus*—ATCC 6538, and *Enterococcus faecalis*—ATCC 25923, respectively, at 24 h post-incubation (hpi) at 37 °C. (**C**–**E**) Inhibition zones (mm) of BV and RD extract (200 μg·mL^−1^) compared with traditional antibiotics against *E. coli*, *S. aureus*, and *E. faecalis*, respectively. Bars and whiskers represent the means and standard deviations (Means ± SDs) of three biological replicates. Different letters signify statistically significant differences between treatments using Tukey’s HSD (*p* < 0.05). CIP: ciprofloxacin (5 µg), AZM: azithromycin (15 µg), S: streptomycin (10 µg), A/S: ampicillin-sulbactam (10/10 µg), and CLR: clarithromycin (15 µg).

Although the three tested bacterial strains were resistant to ampicillin-sulbactam (A/S; 10/10 µg) and compared to conventional antibiotics, both BV (Figure 3A) and RD (Figure 3B) extracts exhibited significant antibacterial activity against the three tested bacterial strains. Briefly, BV and RD extracts exhibited noticeable inhibition zones across all three bacterial strains, with the largest inhibition zones observed for *S. aureus*, suggesting it is the most sensitive strain, whereas both *E. coli* and *E. faecalis* exhibited smaller inhibition zones, indicating less sensitivity (Figure 3A,B).

In agreement with these visual observations, RD extract (200 μg·mL^−1^) showed the highest inhibition zones against *E. coli* (15.0 ± 1.0 mm) when directly compared with standard antibiotics, and was comparable to the most effective traditional antibiotic, ciprofloxacin (5 µg), with no significant differences between them, and followed by BV extract (11.0 ± 1.0 mm) (Figure 3C). On the other hand, RD extract proved to be more effective against *S. aureus* (inhibition zone = 20.0 ± 2.0 mm) than BV extract (13.3 ± 1.2 mm) and all other antibiotics tested (ranged from 8.0 ± 1.0 to 14.7 ± 1.2 mm) (Figure 3D). Additionally, both extracts (BV and RD) showed wide inhibition zones against *E. faecalis* (15.3 ± 0.6 and 18.3 ± 1.5 mm, respectively) similar to those caused by traditional antibiotics, and even were better than azithromycin (AZM; 15 µg) (*p* < 0.0001) (Figure 3E). These differences in the inhibition zones suggest the variability in antimicrobial efficacy between the two extracts and their strain-specific effectiveness.

### 3.4. Effects of BV and RD Extracts on Cellular Morphology of Tested Strains

**Figure 4:**
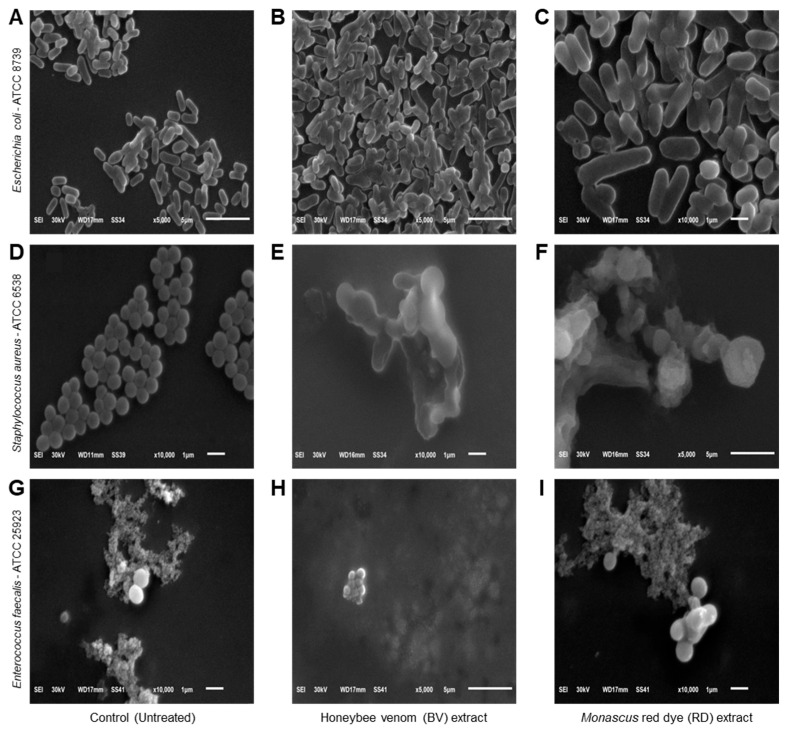
Effects of honeybee venom (BV) and *Monascus* red dye (RD) extracts on cellular morphology of three multidrug-resistant human pathogenic bacteria. (**A**–**C**) Scanning electron microscopy (SEM) micrographs illustrating the cytomorphology of *E. coli* before the treatment (control), after treatment with the BV extract, and after treatment with the RD extract, respectively. (**D**–**F**) SEM-based micrographs of *S. aureus* before the treatment (control), after treatment with the BV extract, and after treatment with the RD extract, respectively. (**G**–**I**) SEM-based micrographs of *E. faecalis* before the treatment (control), after treatment with the BV extract, and after treatment with the RD extract, respectively. The micrographs are presented at varying magnification levels, denoted by the scale bars. Images (**A**,**B**,**F**,**H**) were acquired at 5000× magnification, while micrographs (**C**–**E**,**G**,**I**) were captured at higher magnification (10,000×).

Scanning electron microscopy (SEM) was used to examine the morphological changes in bacterial cells exposed to BV and RD extracts at 200 μg·mL^−1^ compared to untreated controls. Control *E. coli* cells displayed typical rod-shaped morphology with intact, smooth surfaces (Figure 4A). In contrast, extract-treated *E. coli* cells showed severe structural damage, characterized by wrinkled and shriveled surfaces with visible perforations and indentations (Figure 4B,C). Some cells appeared hollow with depleted cellular contents. Control *S. aureus* exhibited characteristic spherical cells in grape-like clusters with uniform, smooth surfaces (Figure 4D). Following exposure to BV (Figure 4E) and RD (Figure 4F) extracts, most cells showed significant morphological alterations including distortion, enlargement, and surface irregularities with visible holes and indentations. A small subset of cells maintained their original morphology with intact surfaces. Untreated *E. faecalis* cells displayed their typical ovoid shape with smooth surfaces, predominantly appearing in diplococcal arrangements (Figure 4G). Treatment with the extracts induced substantial morphological changes, resulting in cell shrinkage and surface damage characterized by multiple small perforations and indentations (Figure 4H,I).

### 3.5. Chemical Characterizations of the BV and RD Extracts

**Figure 5:**
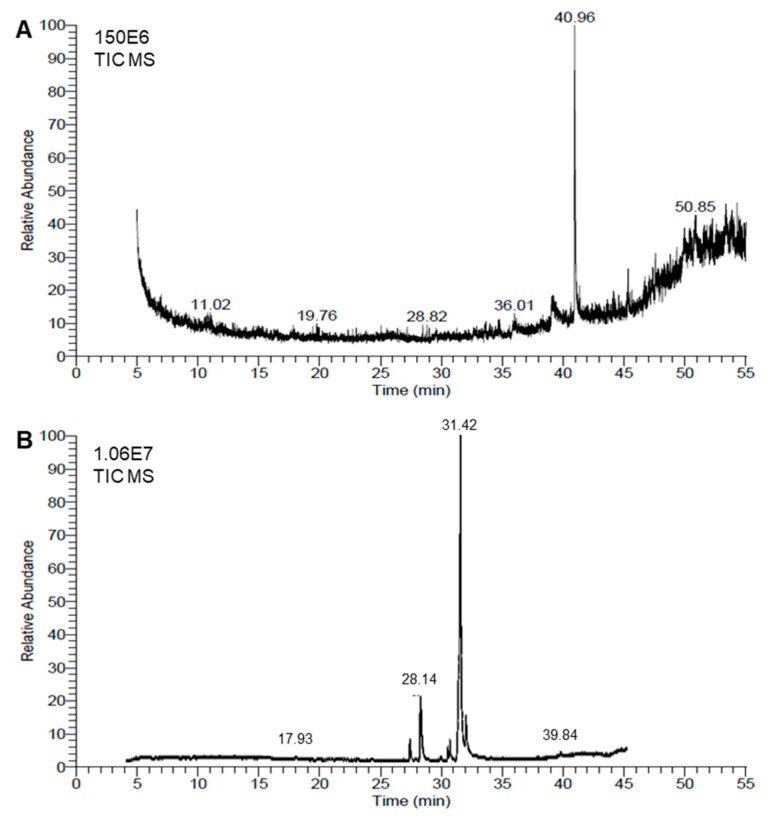
Chemical analyses of honeybee venom (BV) and *Monascus* red dye (RD) extracts using gas chromatography–mass spectrometry (GC–MS) running in the full-scan mode. (**A**,**B**) Representative chromatograms of BV dimethyl sulfoxide (DMSO)-based extract and RD extract, respectively.

Chemical analyses of the honeybee venom (BV) extract and *Monascus* red dye (RD) extract were conducted using the GC–MS method. The resulting chromatograms are presented in Figure 5A,B, respectively. The GC–MS analysis showed that the BV extract was richer than the RD extract, since about 42 metabolites were identified in the BV extract (Table 2) compared with only 23 molecules in the red dye extract (Table 3).

The chemical composition of BV extract exhibited highly diverse structures, including complex polycyclic systems, porphyrins, steroids, and esters. The most abundant compound, 9-Octadecen-1-o (RT = 40.96), accounts for over a quarter of the total peak areas (28.35%), followed by Aralionine (RT = 49.94, 5.16%). Notable compounds include Dotriacontane (RT = 44.56, 1.55%), a long-chain hydrocarbon, and 1,2-Benzenedicarboxylic acid, di-isooctyl ester (RT = 45.34 and 4.45%), a widely used plasticizer. Other significant entries feature bioactive molecules like Astaxanthin (RT = 48.23, 1.6%) and structural components like Flavone 4′-oh,5-oh,7-di-o-glucoside (RT = 47.34, 3.43%) (Figure 5A and Table 2). This diversity highlights a mix of organic and metal–organic compounds with applications across pharmaceuticals, materials, and biochemistry fields.

On the other hand, among the 23 metabolites identified in the extract of RD, fatty acids and their derivatives were dominant, with oleic acid being the most abundant (RT = 31.42, 62.48%). Additionally, high peak-area percentages were noticed for *n-hexadecanoic acid* (RT = 28.14 and 12.76%), *Octadecanoic acid* (RT = 31.87 and 10.38%), and methyl esters such as *Hexadecanoic acid*, *methyl ester* (RT = 27.26 and 2.76%) (Table 2). Other notable compounds include cis-5,8,11,14,17-Eicosapentaenoic acid (0.87%), an omega-3 fatty acid, and 1,25-Dihydroxyvitamin D3 (0.3%), a bioactive vitamin derivative. Collectively, this highlights their applications in antimicrobial, biochemistry, and pharmaceutical fields (Figure 5B and Table 3).

## 4. Discussion

Antimicrobial resistance (AMR) represents a critical global health challenge, characterized by microorganisms’ ability to survive exposure to antimicrobial agents, particularly antibiotics. While AMR occurs naturally, its acceleration has been driven by inappropriate antibiotic use [1]. The magnitude of this crisis is reflected in recent statistics: AMR caused approximately 1.27 million deaths in 2019, with projections indicating potential annual casualties exceeding 10 million by 2050 [2]. The World Health Organization (WHO) has emphasized the risk of AMR developing into a gradual pandemic without significant changes in antibiotic stewardship [39]. This situation has intensified research into natural antimicrobial alternatives [24], with particular attention to honeybee venom (BV) and *Monascus* red dye (RD) as potential therapeutic agents against pathogens.

Chemical characterization of BV through GC–MS analysis revealed several bioactive compounds with therapeutic potential. Key identified molecules included astaxanthin, hycanthone, and fucoxanthin, which demonstrate broad-spectrum antimicrobial activity alongside antiviral, anti-aging, and anticancer properties. These compounds have already found applications in commercial therapeutic products [40]. Additional bioactive components identified in BV, such as tetraneurin-A-diol and dotriacontane, are documented for their antimicrobial and antiviral efficacy [41,42]. The chemical profile of BV showed a distinct influence from the bees’ citrus-based diet, evidenced by the presence of citrus-associated compounds including flavone 4′-OH,5-OH,7-dI-O-Glucoside, dotriacontane, and aralionine [43].

The main compound in BV, melittin, is capable of penetrating the peptidoglycan layer of bacterial cell walls, which may explain its strong antimicrobial activity, particularly against Gram-positive bacteria like *E. faecalis* [44]. BV melittin exhibits its antibacterial activity primarily through membrane-targeted mechanisms. The positively charged peptides strongly interact with the negatively charged membrane phospholipids, leading to membrane destabilization and eventual rupture. This interaction results in the formation of transmembrane pores, compromising bacterial cell integrity [45].

In parallel, the fatty acids present in RD extract demonstrate multiple mechanisms of antimicrobial action. They primarily target cellular bioenergetics by disrupting energy production pathways and interfering with essential enzyme activities and nutrient transport systems. At the membrane level, these fatty acids interact with bacterial phospholipids to form lipid micelle aggregates, which sequester membrane components and severely compromise membrane integrity. This leads to the collapse of the proton gradient, resulting in critical energy losses and eventual cell death [46,47].

Similarly, GC–MS analysis of RD revealed a range of bioactive molecules, including aspidospermidin-17-ol, 1-acetyl-16-methoxy; octanoic acid; hexadecanoic acid methyl ester; and cyclopentaneundecanoic acid. These compounds are known for their broad-spectrum antimicrobial properties and are also recognized for their antiviral, anti-inflammatory, immunomodulatory, and anticancer activities [48,49]. The analysis also indicated a high concentration of free fatty acids, particularly oleic acid, which constituted 62% of the RD extract’s chemical composition. Free fatty acids are well known for their antibacterial properties and are used by organisms to defend against pathogenic bacteria [46].

Our GC–MS analysis identified 23 metabolites in the extract of RD. Among the metabolites, fatty acids and their derivatives were dominant, with oleic acid being the most abundant compound. It was reported previously that one of the most distinguished biological activities of fatty acids (FAs) is their ability to kill or inhibit the growth of microorganisms [50]. However, the direct contributions of fatty acids to antimicrobial activity are poorly understood. It was suggested that the most common target of action is the cell membrane [50,51,52]. For instance, fatty acids negatively affected bacterial viability and biofilm production [51] and disrupted the bacterial membranes in *S. aureus* [52]. This aligns with our SEM observations of membrane damage. Moreover, FAs may increase permeability and succeeding cell lysis, disrupting the electron transport chain and inhibiting enzymatic activity and nutrient intake [50]. Moreover, they can disrupt the microorganisms’ metabolic pathways, inhibit DNA/RNA replication, and affect the expression of virulence genes [50]. Further studies are required to better explain the potential antibacterial role(s) of fatty acids against MDR human pathogenic bacteria.

The minimum inhibitory concentration (MIC) and minimum bactericidal concentration (MBC) results demonstrated strong antibacterial activity of BV and RD extracts against three bacterial strains, with particularly effective results against *S. aureus*. Previous studies have reported similar findings for BV, especially against *E. coli* and *S. aureus*. For example, Maitip et al. [53] observed significant antimicrobial effects of BV against these pathogens, and Bakhiet et al. [54] further confirmed the antibacterial properties of BV against *E. coli* and *S. aureus*. In the current study, we initially tested serial concentrations of the BV and RD extracts (up to 200 μg·mL^−1^) against the three bacterial strains in Mueller–Hinton broth; however, we decided to limit the concentration to the highest concentration (200 μg·mL^−1^), since it showed the highest inhibition based on preliminary experiments. We believe that testing higher concentrations could help identify the plateau of antibacterial efficacy and provide a clearer picture of the full dose–response curve. It is worth mentioning that although the IC_50_ values presented (e.g., *S. aureus*: 1.183, and 3.131 µg.mL^−1^ for BV and RD, respectively) are promising in vitro; however, the translation of these concentrations to in vivo settings requires further investigation. Achieving these concentrations in vivo depends on factors such as bioavailability, pharmacokinetics, and the route of administration. Further investigations are required to explore the effective dose and the potential for these compounds to reach therapeutic levels in clinical settings.

Data on the antibacterial activity of *Monascus* red dye against the specific bacterial strains tested here are limited; however, other studies report similar antibacterial effects of RD, especially against *B. subtilis* ATCC 6633 and *E. coli* MG1655 [55]. Chaudhary et al. [56] also found that RD exhibited antibacterial activity against *B. cereus* and *E. coli*.

To assess the potential of BV and RD as alternatives to traditional antibiotics, their antibacterial activities were compared to five commonly used antibiotics using the disc diffusion method. The bacterial strains were sensitive to four of the antibiotics but resistant to ampicillin-sulbactam. RD showed the largest inhibition zone against *S. aureus* (20 ± 0.22 mm), followed by a notable inhibition zone against *E. faecalis* (18 ± 0.15 mm). BV also exhibited significant antibacterial effects, with the largest inhibition zone observed against *E. faecalis* (15 ± 0.22 mm), followed by *S. aureus* (13 ± 0.15 mm). Among the antibiotics, ciprofloxacin (CIP) produced the largest inhibition zones, while azithromycin (AZM) had the smallest zones. These findings are consistent with previous studies [57,58]. Overall, both BV and RD exhibited potent antibacterial effects, comparable to several antibiotics tested.

Microscopic examination of the bacterial cells provided additional insight into the antibacterial mechanisms of BV and RD. Scanning electron microscopy (SEM) revealed significant structural changes in the bacterial cells treated with these extracts. Treated strains showed rough, shrunken, and wrinkled surfaces compared to the smooth, well-defined surfaces of untreated control cells. These results suggest that BV and RD disrupt the bacterial cell membrane, increasing permeability and leading to ATP loss.

We believe that evaluating the cytotoxicity of BV and RD extracts is crucial before proposing them as potential therapeutic agents. Although cellular toxicity and biological activities of BV were well-reported previously [59,60,61,62], our knowledge about the cytotoxicity of *Monascus* fermentation products is still limited [63]. For example, BV induced cell death in normal human lymphocytes and HL-60 cells in a time-dependent manner till 24 h post-treatment (hpt); however, these cytotoxic effects ended thereafter, which may have been due to the half-life of BV [59]. Likewise, BV showed cytotoxic, genotoxic, and mutagenic potentials to HepG2 cells at 3 hpt; however, low concentrations of 0.1, 0.05, and 0.01 μg·mL^−1^ were not cytotoxic [60]. Similarly, BV application exhibited significant antiproliferative and cytotoxic effects on several tumorigenic cell lines and nontumorigenic cells [61]. Likewise, it displayed antibacterial activities against MDR human pathogens such as Extended Spectrum Beta-Lactamases producing *E. coli* and vancomycin-resistant *Enterococcus faecium* [61]. It is worth mentioning that detoxification of BV via hydrolyzation of melittin, the main metabolite of BV, significantly decreased its cytotoxicity and allergenic activity in MCF 10A and RAW 264.7 cells [62].

On the other hand, it was reported previously that the presence of the secondary metabolite of *Monascus* species, citrinin (CTN), in fermentation products, is a potential threat to public health. CTN was detected in lipid extracts of *Monascus* species but was not found in aqueous extracts [63]. Accordingly, when human embryonic kidney cells (HEK293) were incubated for 72 h with *Monascus* extracts, the concentrations causing 50% cell death by all lipid extracts were in the range of 1.8–4.7 mg.mL^−1^, whereas aqueous extracts showed a lower cytotoxicity [63]. Collectively, these findings suggest that aqueous extracts of BV and RD have promising antibacterial properties and might be potential alternatives to conventional antibiotics. However, future investigations are required to assess the cytotoxicity of both extracts and to determine safe and effective dosage ranges.

## 5. Conclusions

In response to the global challenge of antimicrobial resistance (AMR) and declining antibiotic efficacy, this study investigated novel antimicrobial agents from natural sources. We evaluated two natural products: honeybee venom (BV) collected from honeybee colonies at the Faculty of Agriculture apiary, Tanta University, and red dye (RD) extracted from *Monascus purpureus*. GC–MS analysis of both extracts revealed diverse bioactive metabolites with documented biological activities, particularly antimicrobial properties. Both extracts demonstrated robust antibacterial activity against all tested bacterial strains, with MIC and MBC values indicating potent antimicrobial effects. Notably, both BV and RD produced larger inhibition zones compared to conventional antibiotics, suggesting their potential as therapeutic alternatives. Nevertheless, while the current study focused on MDR bacterial cultures to establish baseline antimicrobial activity and mechanisms of BV and RD, evaluating their efficacy and performance in complex systems such as mixed microbial communities or biofilms in biofilms is required, which is more representative of clinical and environmental settings. Additionally, further studies on evaluating the synergistic effects of RD extracts with existing traditional antibiotics could enhance their applicability against MDR pathogens. Finally, further pharmacological studies are required to elucidate their precise mechanisms of action, evaluate potential toxicity and side effects, determine optimal dosing regimens, develop standardized pharmaceutical formulations, and assess stability and shelf-life parameters.

## Figures and Tables

**Table 1:** The half-maximal inhibitory concentration (IC_50_; μg·L^−1^) results of honeybee venom (BV) and *Monascus* red dye (RD) extracts against multidrug-resistant human pathogenic bacteria.

Bacterial Strain	IC_50_ (μg·mL^−1^)	95% Confidence Interval		Overall Model Fit			
		Lower	Upper	χ^2^	*p*-Value	Cox & Snell R^2^	Nagelkerke R^2^
Honeybee venom (BV) extract							
*E. coli* ATCC 8739	5.340	3.080	8.929	501.384	<0.0001	0.3943	0.5287
*S. aureus* ATCC 6538	1.183	0.524	2.069	290.859	<0.0001	0.2524	0.3696
*E. faecalis* ATCC 25923	14.921	7.181	32.692	615.206	<0.0001	0.4595	0.6186
*Monascus* red dye (RD) extract							
*E. coli* ATCC 8739	40.876	10.632	491.037	567.824	<0.0001	0.4329	0.6256
*S. aureus* ATCC 6538	3.131	2.729	3.592	846.946	<0.0001	0.5709	0.7843
*E. faecalis* ATCC 25923	18.758	6.201	61.248	770.928	<0.0001	0.5371	0.7303

**Table 2:** Retention times (min), peak area percentages (%), molecular formulas, and molecular masses of detected metabolites in the extract of honeybee venom (BV) using gas chromatography–mass spectrometry (GC–MS) running in full-scan mode.

No	Compound	RT	%Area	Molecular Formula	Mass
1	5-HCyclopropa[3,4]benz[1,2-e]azulen-5-one,4,9,9-atris(acetyloxy)3[(acetyloxy)methyl]1,1a,1b,4,4a,7a,7b,8,9,9a-decahydro-4a,7-bdihydroxy1,1,6,8-tetramethyl	6.88	0.78	C_28_H_36_O_11_	549
2	2,2Bis[4[(4,6dichloro1,3,5triazin2yl)oxy]phenyl]1,1,1,3,3,3hexafluoropropane	7.82	0.82	C_21_H_8_Cl_4_F_6_N_6_O_2_	632
3	Hycanthone	9.26	0.81	C_20_H_24_N_2_O_2_S	356
4	4,5,6,7Tetrakis(pchlorophenoxy)1,2diiminoisoindoline	10.79	1.11	C_32_H_19_Cl_4_N_3_O_4_	10.79
5	Copper tetraphenylporphyrin	11.11	0.9	C_44_H_28_CuN_4_	676
6	3,4,10,11tetrakis(Dimethylamino)7,14bis(trifluoromethyl)7,14epoxydinaphtho[1,8ab:1′,8′ef]cyclooctane	11.79	0.78	C_32_H_32_F_6_N_4_O	603
7	Decanoicacid,1,1a,1b,4,4a,5,7a,7b,8,9decahydro4a,7bdihydroxy1,1,6,8tetramethyl5oxo3[[(1oxodecyl)oxy]methyl]9aHc yclopropa[3,4]benz[1,2e]azulene9,9adiylester	11.95	1.05	C_50_H_82_O_9_	827
8	2-Myristynoyl pantetheine	17.83	1.2	C_25_H_44_N_2_O_5_S	484
9	2,4bis(áchloroethyl)6,7bis[ámethoxycarbonylethyl]8formyl1,3,5trimethylporphyrin	19.76	1.03	C_36_H_38_Cl_2_N_4_O_5_	677
10	Tetraneurin-A-diol	32.66	0.83	C_15_H_20_O_5_	280
11	Tristrimethylsilyl ether derivative of 1,25-dihydroxyvit amine d2	33.55	1.16	C_37_H_68_O_3_Si_3_	645
12	Pregn-4-ene-3,11,20trione,6,17,21-tris[(trimethylsilyl)oxy]-,3,20-bis(O-methyloxime), (6á)-	33.65	0.91	C_32_H_58_N_2_O_6_Si_3_	651
13	16-Oxapentacyclo [13.2.2.0(1,13).0(2,10).0(5,9)] nonadecane	35.58	0.74	C_22_H_34_D_2_O_3_	346
14	*trans*-2-Phenyl-1,3-dioxolane4methyloctadec-9,12,15-trienoate	35.88	0.88	C_28_H_40_O_4_	440
15	2-Cyclohexyl-4a,7-dimethyl-3,4,4a,5,6,8a-hexahydro-2H-benzo[e][1,2]oxazine-3-carbonitrile	38.28	0.81	C_17_H_26_N_2_O	274
16	Butanoicacid,4-chloro,1,1a,1b,4,4a,5,7a,7b,8,9-decahydro-4a,7b-dihydroxy-3-(hydroxymethyl)-1,1,6,8-tetramethyl-5-oxo-9a-Hcyclopropa[3,4]benz[1,2-e]azulene9,9a-diylester	39.03	0.76	C_28_H_38_Cl_2_O_8_	573
17	4-(-1-hydroxyethyl)-1,6,7-tris-(2-methoxycarbonylethyl)-2,3,5,8-tetramethylporphyrin	39.16	1.23	C_38_H_44_N_4_O_7_	668
18	9-Octadecen-1-ol,(Z)-(CAS)	40.96	28.35	C_18_H_36_O	268
19	6-C-Xylosyl-8-C-glucosylapigenin-permethylated derivative	42.38	1.43	C_33_H_36_O_17_	704
20	5á-Pregnan-20-one,3à,11á,17,21-tetrakis(trimethylsiloxy)-,O-methyloxime	43.64	1.2	C_34_H_69_NO_5_Si_4_	684
21	(22S)-21-Acetoxy-6à-,11ádihydroxy16à,17à-propylmethylenedioxypregna-1,4-diene-3,20-dione	44.13	1.15	C_27_H_36_O_8_	488
22	N,N’-Dicyclohexyl-1,7-dipyrrolidinylperylene-3,4:9,10-tetracarboxylicacid bisimide	44.2	0.73	C_44_H_44_N_4_O_4_	692
23	Dotriacontane (CAS)	44.56	1.55	C_32_H_66_	450
24	Isochiapin B	44.81	1.01	C_19_H_22_O_6_	346
25	1,2-Benzenedicarboxylic acid, di isooctyl ester(CAS)	45.34	4.45	C_24_H_38_O_4_	390
26	(5,10,15,20-tetraphenyl[2-(2)H1]prophyrinato)zinc(II)	45.65	1.14	C_44_H_27_DN_4_Zn	677
27	3,5,9-Trioxa-5-phosphaheptacos-18-en-1-aminium,4-hydroxy-N,N,N-trimethyl-10-oxo-7-[(1-oxo-9-octadecenyl)oxy]-,hydroxide, inner salt,4-oxide,(R)	46.64	1.35	C_44_H_84_NO_8_P	786
28	3-Hydroxy-1-(4{13-[4-(3-hydroxy-3-phenylacryloyl)phenyl]tridecyl}-phenyl)-3-phenylprop-2-en-1-one	46.69	1.06	C_43_H_48_O_4_	628
29	Pregn-4-ene-3,20-dione, 17,21-dihydroxy-,bis(Omethyloxime)	46.73	0.88	C_23_H_36_N_2_O_4_	404
30	Corynan-17-ol,18,19-didehydro-10-methoxy-,acetate (ester)	47.09	2.19	C_22_H_28_N_2_O_3_	368
31	Flavone4′-oh,5-oh,7-di-o-glucoside	47.34	3.43	C_27_H_30_O_15_	594
32	4,25-Secoobscurinervan-21-deoxy-16-methoxy-22-methyl-,(22à)-(CAS)	47.58	3.89	C_23_H_32_N_2_O_2_	368
33	Fucoxanthin	47.86	1.06	C_42_H_58_O_6_	658
34	4H-Cyclopropa[5′,6′]benz[1′,2′:7,8]azuleno[5,6-b]oxiren-4-one,8,8abis(acetyloxy)-2a-[(acetyloxy)methy-l]1,1a,1b,1c,2a,3,3a,6a,6b,7,8,8a-dodecahydro-3,3a,6b-trihydroxy-1,1,5,7-tetramethyl-	48.08	3.07	C_26_H_34_O_11_	522
35	Astaxanthin	48.23	1.6	C_40_H_52_O_4_	596
36	Benzene,2-(1-decyl-1-undecenyl)-1,4-dimethyl-(CAS)	48.29	1.72	C_29_H_50_	398
37	9-Octadecenoicacid,(2-phenyl-1,3-dioxolan-4-yl)methyl ester, cis-(CAS)	48.35	1.77	C_28_H_44_O_4_	444
38	(2-hydroxy-5,10,15,20-tetraphenylporphinato)zinc(II)	48.53	2.81	C_44_H_28_N_4_OZn	694
39	Ethyl iso-allocholate	49.02	1.46	C_26_H_44_O_5_	436
40	Tetraphenylporphyrinat odibromotitanium(IV)	49.34	2.07	C_44_H_28_Br_2_N_4_Ti	820
41	Stigmast-5-en-3-ol,(3á,24S)-(CAS)	49.83	1.19	C_29_H_50_O	414
42	Aralionine	49.94	5.16	C_34_H_38_N_4_O_5_	582

**Table 3:** Retention times (min), peak area percentages (%), molecular formulas, and molecular masses of detected metabolites in *Monascus* red dye (RD) extract using gas chromatography–mass spectrometry (GC–MS) running in full-scan mode.

No	Compound	RT	%Area	Molecular Formula	Mass
1	Aspidospermidin-17-ol,1-acetyl-16-methoxy-	5.23	0.32	C_22_H_30_N_2_O_3_	370
2	Ethanimidothioic acid, 2-(dimethylamino)-n-[[(methylamino)carbonyl]oxy]-2-oxo-, methyl ester	5.35	0.55	C_7_H_13_N_3_O_3_S	219
3	Octanoic acid, 7-oxo-	5.56	0.2	C_8_H_14_O_3_	158
4	Hexadecanoic acid, methyl ester	27.26	2.76	C_17_H_34_O_2_	270
5	n-Hexadecanoic acid	28.14	12.76	C_16_H_32_O_2_	256
6	17-Octadecynoic acid, TMS derivative	29.83	0.52	C_21_H_40_O_2_Si	352
7	9,12-Octadecadienoic acid, methyl ester, (E,E)-	30.38	1.55	C_19_H_34_O_2_	294
8	9-Octadecenoic acid (Z)-, methyl ester	30.56	2.1	C_19_H_36_O_2_	296
9	Oleic Acid	31.42	62.48	C_18_H_34_O_2_	288
10	Octadecanoic acid	31.87	10.38	C_18_H_36_O_2_	282
11	9-Octadecenoic acid (z)	32.25	0.72	C_18_H_34_O_2_	282
12	9,12-Octadecadienoyl chloride, (Z,Z)	32.32	0.69	C_18_H_31_ClO	298
13	Hi-oleic safflower oil	32.46	0.44	C_21_H_22_O_11_	450
14	2-Aminoethanethiol hydrogen sulfate (ester)	32.53	0.38	C_2_H_7_NO_3_S_2_	157
15	13,16-Octadecadienoic acid, methyl ester	32.6	0.4	C19H34O2	294
16	17-Octadecynoic acid	32.72	0.54	C_18_H_32_O_2_	280
17	Cyclopentaneundecanoic acid	32.84	0.37	C_16_H_30_O_2_	254
18	8,11,14-Eicosatrienoic acid, (Z,Z,Z)-	33.03	0.37	C_20_H_34_O_2_	306
19	cis-5,8,11,14,17-Eicosapentaenoicacid	39.65	0.87	C_20_H_30_O_2_	302
20	9,12,15-Octadecatrienoic acid,	40.88	0.22	C_27_H_52_O_4_Si_2_	496
21	Trideuteriomethyl10-epoxy-7-ethyl-3,11-dimethyltrideca-2,6-dienoate	41.13	0.18	C_18_H_27_D_3_O_3_	297
22	1,25-Dihydroxyvitamin D3, TMS derivative	41.41	0.3	C_30_H_52_O_3_Si	488
23	Cholest-5-en-3-yl(9z)-9-octadecenoate	41.51	0.25	C_45_H_78_O_2_	650

## Data Availability

The original contributions presented in the study are included in the article, and further inquiries can be directed to the corresponding authors.
